# Preclinical modeling of myelodysplastic syndromes

**DOI:** 10.1038/leu.2017.172

**Published:** 2017-06-30

**Authors:** K Rouault-Pierre, S A Mian, M Goulard, A Abarrategi, A Di Tulio, A E Smith, A Mohamedali, S Best, A-M Nloga, A G Kulasekararaj, L Ades, C Chomienne, P Fenaux, C Dosquet, G J Mufti, D Bonnet

**Affiliations:** 1 Haematopoietic Stem Cell Laboratory, The Francis Crick Institute, London, UK; 2 King’s College London School of Medicine, Department of Haematological Medicine, London, UK; 3 INSERM, UMRS1131–University Paris Diderot, Saint Louis Hospital, Paris, France; 4 King’s College Hospital, Department of Haematology, London, UK; 5 Senior Haematology Department, Saint Louis Hospital, APHP, Paris, France; 6 Cell Biology Department, Saint Louis Hospital, APHP, Paris, France

## Abstract

Myelodysplastic syndromes (MDS) represent a heterogeneous group of hematological clonal disorders. Here, we have tested the bone marrow (BM) cells from 38 MDS patients covering all risk groups in two immunodeficient mouse models: NSG and NSG-S. Our data show comparable level of engraftment in both models. The level of engraftment was patient specific with no correlation to any specific MDS risk group. Furthermore, the co-injection of mesenchymal stromal cells (MSCs) did not improve the level of engraftment. Finally, we have developed an *in vitro* two-dimensional co-culture system as an alternative tool to *in vivo*. Using our *in vitro* system, we have been able to co-culture CD34^+^ cells from MDS patient BM on auto- and/or allogeneic MSCs over 4 weeks with a fold expansion of up to 600 times. More importantly, these expanded cells conserved their MDS clonal architecture as well as genomic integrity.

## Introduction

Myelodysplastic syndromes (MDS) are a heterogeneous group of clonal hematopoietic stem cell disorders with diverse phenotypes, characterized mainly by ineffective hematopoiesis and bone marrow (BM) morphological dysplasia.^1, 2, 3^ The phenotypic heterogeneity and the highly variable prognosis of MDS patients make it difficult to classify the disease subtype and predict the survival as well as likelihood of transformation to leukemia. It is important to note that one-third of patients with MDS progress to acute myeloid leukemia, whereas the remaining two-thirds evolve from low-risk to high-risk disease.

Over the past decade, there has been significant progress in understanding the molecular pathogenesis underlying the MDS^4, 5, 6, 7^ with studies reporting how self-renewing hematopoietic stem cells continuously acquire somatic aberrations, and although most of them are passenger mutations, some ‘potent mutations’ can constitute a reservoir of preleukemic stem cells.^8, 9^ As more genetic data are gathered, there is an increased need to understand the tumor’s evolutionary history using both longitudinal genomic information and preclinical modeling. Moreover, the dynamics of interactions between subclones, each with their own superimposed developmental hierarchy, whether they compete or are co-dependent upon each other and hence coordinate clonal evolution, needs to be elucidated. Notably, patient-derived xenograft models offer the most advanced preclinical opportunity to capture the complexities of such malignancies.^8, 9^ A number of different animal models have been proposed but the more promising to date are the NSG and the NSG-S (humanized with stem cell factor (SCF), granulocyte-macrophage colony-stimulating factor (GM-CSF) and interleukin-3 (IL-3)) immunodeficient mice.^10, 11^

Here, we have used BM cells from 38 MDS patients (low–intermediate- and high-risk patients) to generate a preclinical *in vivo* and i*n vitro* model that can be used to study clonal evolution and test targeted therapies. We have used NSG and NSG-S mice to assess engraftment potential of MDS samples. Furthermore, using high-depth sequencing, we have confirmed that the MDS clonal population had engrafted in our mice. Finally, to overcome the limitations of the low recovery of cells following xenotransplantation, we have developed an *in vitro* two-dimensional (2D) co-culture system allowing expansion of MDS clones. Using next-generation single-nucleotide polymorphism arrays, we have demonstrated that this *in vitro* co-culture system maintains the genomic landscape of MDS disease BM.

## Materials and methods

### Patients and samples

Patient samples (*n*=38) were received from King’s College London Haemato-Oncology Tissue Bank under research ethics protocol (08/H0906/94) and from Hospital St Louis (France) under ethical approval IRB00006477, opinion number 13-027. Demographic and clinical characteristics of the studied patients are detailed in Table 1. All patients were risk stratified according to IPSS (International Prognostic Scoring System) categories covering all risk groups: low–intermediate- to high-risk groups (Table 1). The clinical variables, FAB (French–American–British), WHO (World Health Organization() subtype and the prognostic risk of all patients, were ascertained at the time of sample collection. Mutation profile of the primary patient BM cells was obtained by whole-exome sequencing or King’s College London myeloid gene-mutation panel (Supplementary Table S1).

### Xenotransplantation

NOD/SCID/IL2rγ^−/−^ (NSG) mice and NOD/SCID/IL2rγ^−/−^/IL-3/GM-CSF/SCF (NSG-S) mice were a kind gift of Dr Leonard Shultz (The Jackson Laboratory, Bar Harbor, ME, USA). All animal experiments were performed in accordance to Home Office and CRICK guidelines. Before transplantation, mice received a sublethal dose of radiation (330–375 cGy) from a cesium-137 source. Direct intra-BM injection was performed in the tibia or femur with 1 × 10^5^ to 2 × 10^5^ BM CD34^+^ cells (with or without mesenchymal stromal cells (MSCs)) or with 1 × 10^6^ CD3^+^ depleted mononuclear cells (MNCs; with or without MSCs, 1:1 ratio) from patients and/or normal controls. Engraftment was assessed at the time of killing (12–18 weeks) and the BM (pooled femurs, tibias, pelvis) was immunophenotyped by the presence of mCD45, hCD45, hCD33, hCD19 and hCD3 (BD Biosciences, Oxford, UK) cell populations. Live cells were stained and sorted on hCD45 phenotype using FACS Aria SORP (BD Biosciences). Sorted cells were washed in phosphate-buffered saline and harvested in order to later perform genomic analysis.

### Long-term culture-initiating cell (LTC-IC) assay

LTC assays were performed by plating 1 × 10^3^ CD34^+^ BM cells in duplicate on irradiated MS-5 murine stromal cells and/or autologous/allogeneic MSCs using Myelocult H5100 (StemCell Technologies, Vancouver, BC, Canada) in the presence of cytokines (20 ng/ml SCF, 20 ng/ml IL-3 and 20 ng/ml thrombopoietin (TPO) from PeproTech, London, UK). After 4 weeks, hCD45^+^ cells were isolated using EasySep Human CD45 Depletion Kit (StemCell Technologies, cat. no. 18259). Isolated CD45^+^ cells were washed in phosphate-buffered saline, counted and then plated into methylcellulose as mentioned below. The rest of the cells were harvested and stored as cell pellet for future use for genomic and single-nucleotide polymorphism assays. Images of LTC were acquired with a Zeiss (Cambridge, UK) AxioVert 40 CFL microscope using 5 × lens and connected to Canon (Melville, NY, USA) PowerShot A640 digital camera.

### Colony-forming cell

A total of 500 cells from the primary BM CD34^+^ enriched fraction were plated in duplicate in 0.5 ml in 24-well plates with MethoCult H4434 (StemCell Technologies). Similarly, 1 × 10^5^ CD45^+^ cells recovered from LTC were plated in duplicate in 35 mm dishes in 1 ml of MethoCult H4435 (StemCell Technologies). Assay was performed under hypoxic conditions (3% O_2_). After day 14 of culture, the numbers of colonies were scored.

Further information is available in the Supplementary Methods.

## Results and discussion

**Figure 1:**
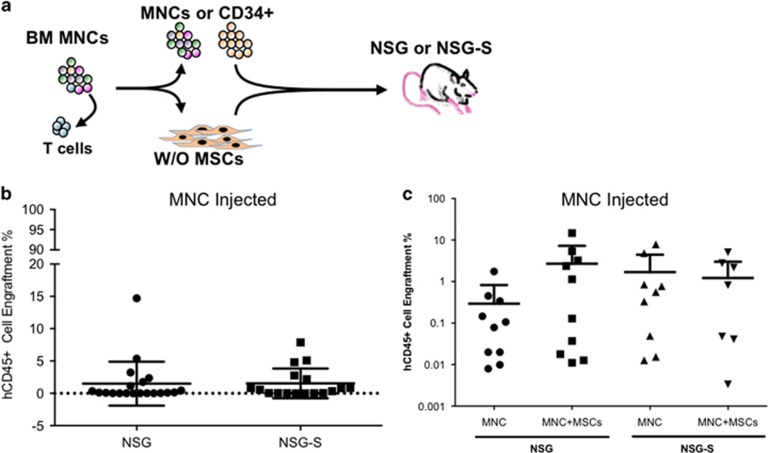
Xenotransplantation of MDS BM MNCs. (**a**) Schematic representation of intrabone injection in NSG/NSG-S mice with or without MSCs. (**b**) hCD45^+^ engraftment in total mouse BM at the time of killing from NSG and NSG-S mice injected with patient MNCs (patients *n*=11; NSG mice *n*=20; NSG-S mice *n*=17). hCD45 ⩾0.01% in total mouse BM was considered as successful human cell engraftment. (**c**) hCD45^+^ engraftment in total mouse BM at the time of killing from NSG and NSG-S mice injected with patient MNCs±MSCs (autologous/allogeneic), (patients *n*=5 (MDS 36, 37, 38, 39 and 49)); MNCs alone NSG mice *n*=10; MNCs+MSCs NSG mice *n*=10; MNC alone NSG-S mice *n*=9; MNCs+MSCs NSG-S mice *n*=9).

Here we have tested the engraftment potential of MDS in NSG and/or NSG-S immunodeficient mouse models. Initially, we choose to screen 11 patients in NSG and/or NSG-S mice by injecting 1 × 10^6^ BM patient MNCs (CD3^+^ depleted) and then treating with OKT3 before injection as described in previous studies.^12^ After 18 weeks, engraftment levels based on human CD45^+^ cells (hCD45^+^) harvested from engrafted mice ranged between 0.01 and 15%. Interestingly, no difference was observed in the level of engraftment between the NSG and NSG-S mice (Figures 1a and b).

Next, we decided to determine whether patient-derived MSCs can help to improve the engraftment of MDS BM cells in NSG as well as in NSG-S mice as has been previously suggested.^11, 13^ Our results obtained from mice that were injected with 1 × 10^6^ patient MNCs (CD3^+^ depleted cells) along with 0.5 × 10^6^ MSCs (autologous or allogeneic) did not show any improvement in engraftment levels in both mouse models (Figure 1c).

**Figure 2:**
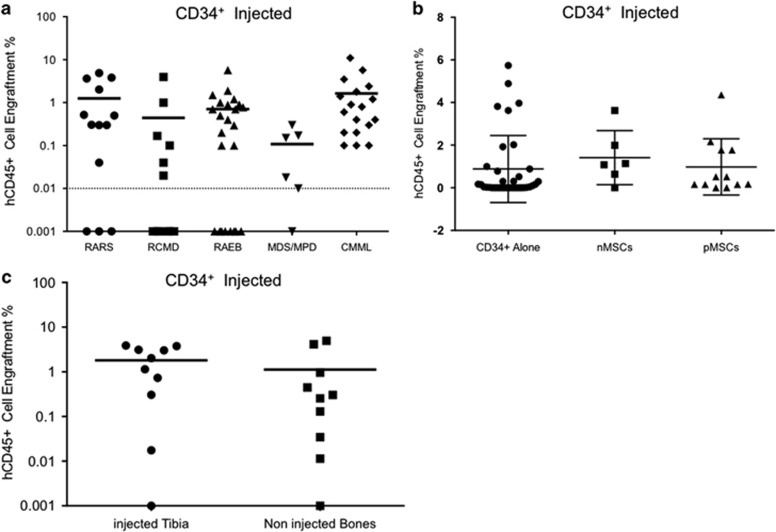
Xenotransplantation of MDS BM CD34^+^ cells. (**a**) Percentage of hCD45^+^ engraftment observed in NSG mice injected with CD34^+^ cells from various MDS WHO subtypes (patients *n*=22; NSG mice *n*=74). hCD45 ⩾0.01% in total mouse BM was considered as successful human cell engraftment. (**b**) hCD45^+^ engraftment in total mouse BM at the time of killing from NSG mice injected with patient CD34^+^±MSCs (autologous/allogeneic) (CD34^+^ alone *n*=35 mice and patients *n*=22, CD34^+^+nMSCs *n*=6 mice and patients *n*=6; CD34^+^+pMSCs *n*=12 mice and patients *n*=6). nMSCs, normal healthy donor MSCs, pMSCs, autologous patient MSCs. (**c**) Comparison of hCD45^+^ engraftment observed in injected bone vs noninjected bones from NSG mice transplanted with patient CD34^+^+MSCs (mice *n*=10, patients *n*=6 (MDS 6, 9, 17, 18, 20 and 27).

In order to determine whether enrichment of malignant stem/progenitor cells can improve the engraftment, we isolated the CD34^+^ cells from 22 patient BM and injected cells ranging from 1 × 10^5^ to 2 × 10^5^ cells into NSG mice (Supplementary Figure S1). The hCD45^+^ engraftment levels measured at the time of killing of mice ranged between 0.01 and 12%. Surprisingly, our data show that the level of engraftment is patient specific (Supplementary Figure S1) and not dependent on the MDS WHO subtype (Figure 2a). We next tested whether injecting CD34^+^ along with autologous or allogeneic MSCs (from healthy BM donors) could improve the level of engraftment. Therefore, we injected 1–2 × 10^5^ CD34^+^ cells with or without 1–2 × 10^5^ MSCs (autologous patient MSCs and normal healthy donor BM MSCs). Interestingly, no difference was observed in the engraftment of hCD45^+^ cells between mice who received CD34^+^ cells alone or with MSCs (Figure 2b). As cells were injected intrabone and MSCs have been previously suggested to provide ‘niche units’^11^ that helps in enhancing the engraftment of MDS BM cells, we assessed the level of engraftment in the injected bone as well as the rest of the bones (tibia, femurs and pelvis). The level of hCD45^+^ cells was similar between all the analyzed bones at week 18 (Figure 2c), suggesting that engraftment potential of the MDS BM cells might be independent of the presence of human MSCs.

To confirm the nature of the human engrafted cells recovered from mice, targeted mutational analysis was performed on xenografted hCD45^+^ cells (where available). Our sequencing analysis confirmed the presence of the malignant MDS clone(s) in all the mice engrafted with human cells (Supplementary Table S2). Apart from two cases (MDS1 and MDS38), mutant allele burden of MDS-related gene mutations were maintained (compared with day 0 patient BM cells) in the xenografts, irrespective of whether the mice were injected with MNCs or CD34^+^ cells.

**Figure 3:**
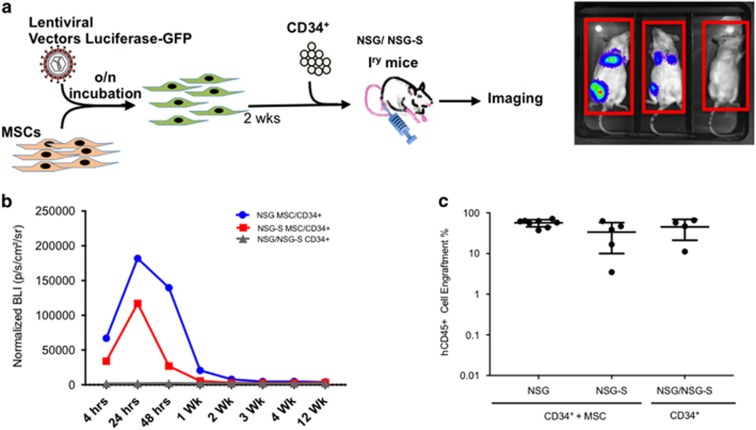
Xenotransplantation of cord blood (CB) CD34^+^ cells and tracking of hMSCs. (**a**) Schematic representation of lentiviral transduction of human MSCs with GFP–luciferase vector, followed by intrabone injection in NSG/NSG-S mice and *in vivo* imaging. (**b**) Bioluminescence plot showing the photons emitted from luciferase-expressing MSCs over the 12-week period (CD34^+^+MSCs in NSG *n*=8; CD34^+^ alone in NSG *n*=2; CD34^+^+MSCs NSG-S *n*=5; CD34^+^ alone NSG-S *n*=2). (**c**) hCD45^+^ engraftment in total mouse BM at the time of killing from NSG and NSG-S mice injected with cord blood CD34^+^±MSCs (CB CD34^+^+MSCs, NSG=8, NSG-S=5; CB CD34^+^, NSG/NSG-S=4).

In order to evaluate the kinetics of human MSC (hMSC) engraftment *in vivo*, we transduced MSCs from healthy donors with a bicistronic vector coexpressing green fluorescent protein (GFP) and luciferase (with a transduction efficiency ranging from 80 to 90%) and coinjected them with CD34^+^ cells isolated from cord blood (Figure 3a) into either NSG and NSG-S mice (*n*=13). Control mice (*n*=4) were injected only with cord blood CD34^+^ cells. Bioluminescence was assessed at 4, 24 and 48 h after intra-BM (right femur) injection and then once a week over a period of 4 weeks as well as at 12 weeks (Figures 3b and c). Strikingly, we were not able to detect any hMSCs by bioluminescence 1 week after injection. At week 12, we collected injected bones from 13 mice transplanted with MSC+CD34^+^ cells and performed immunofluorescence staining against the GFP^+^ protein expressed by our transduced hMSCs. Although we could not detect hMSCs by bioluminescence, we observed a few GFP^+^ cells scattered in the long injected bone in 2/13 mice (Supplementary Figure S2). We therefore analyzed the adjacent noninjected bone (right tibia) and looked for GFP^+^ cells. Notably, we were not able to detect any GFP^+^ cells in the tibia from the two mice that had residual GFP^+^ cells in injected femur, thereby confirming that MSCs do not have colonization capacity (Supplementary Figure S3). Next, we sought to determine whether the level of cord blood CD34^+^ engraftment would increase by the coinjection of hMSCs. Interestingly, the percentage of human cell engraftment was similar in mice that received CD34^+^ alone versus CD34^+^+hMSCs (hCD45 45%±24 versus 48%±35 respectively; (Figure 3c and Supplementary Table S3). Taken together, these results suggest that hMSCs even injected intrabone do not persist for >1 week in NSG or NSG-S mice, and thus potentially explain why their coinjection with patient MNCs or CD34^+^ cells do not affect the long-term engraftment of these cells. However, it is possible that the hMSCs might be providing a supportive niche during the initial establishment of the graft as has been suggested by Meydouf *et al.*^11^ (first week post transplant) and this effect, if any, subsides over time (killed 12–18 weeks post injection; Figures 2b and 3c and Supplementary Table S3).

Our study and other previously published reports have shown that it is possible to engraft MDS patient samples into immunodeficient mice, and characterize the nature of the MDS-initiating cells as well as clonal evolution overtime;^8, 9, 10^ however, the low level of engraftment generally observed makes these models impractical to use reliably for screening of potential novel targeted therapies. Therefore, we focused our attention on developing an *ex vivo* 2D culture model. We used autologous (and/or allogeneic) MSCs and CD34^+^ cells isolated from patients BM, therefore providing a unique system to study both the stroma and hematopoietic cells.

Patients (*n*=9) from all WHO MDS subtypes (RARS (refractory anemia with ringed sideroblasts), RCMD (refractory cytopenia with multilineage dysplasia), RAEB1 (refractory anemia with excess blasts-1) and RAEB-2 (refractory anemia with excess blasts-2)) were chosen for analysis using our 2D co-culture system. Initially, fresh patient BM CD34^−^ cells were used to expand the MSCs, whereas part of the CD34^+^ fraction was used for single-cell clonogenic CFC assay (Supplementary Figure S4a). Later on, once MSC cultures were established, MDS patient CD34^+^ cells were used to generate LTC-ICs on autologous or allogeneic MSCs.

**Figure 4:**
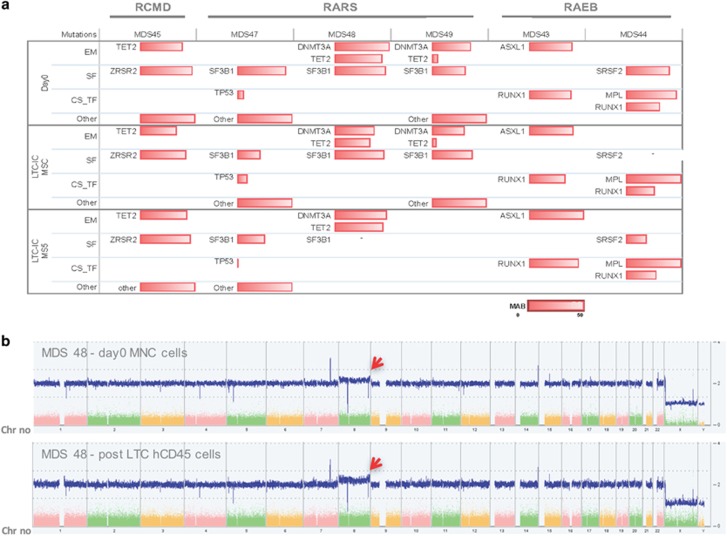
Sequencing and single-nucleotide polymorphism (SNP) karyotyping of *in vitro* MDS samples. (**a**) Mutational analysis of day 0 BM total nucleated cells or CD34^+^ cells and hCD45^+^ cells retrieved after LTC (MSCs and/or MS5; patients *n*=6; ‘-’ not available). (**b**) SNP karyotyping showing the maintenance of MDS48 clonality between pre- and post-culture. Presence of a trisomy-8 subclone (see red arrow) in both day 0 and post culture on MS-5.

**Figure 5:**
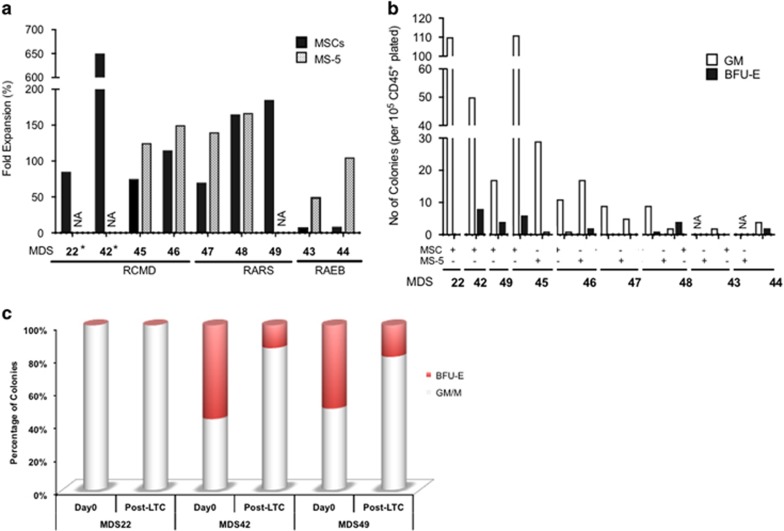
The 2D *in vitro* modeling of MDS. (**a**) Fold expansion of cells observed after LTC of patient CD34^+^ cells grown on MSCs and/or MS5 for a period of 4 weeks (patients *n*=9; NA, not available). (**b**) Total number of colonies (per 1 × 10^5^ CD45^+^ seeded cells) generated following the LTC (obtained from (**a**)) for patients across various MDS WHO subtypes (patients *n*=9). (**c**) Comparison of burst forming unit- erythroid/granulocyte-macrophage colony ratio from primary CD34^+^ cells versus post-LTC (patients *n*=3; NA, not available).

First, it is important to note that the MDS clone as determined by the presence of the gene mutation(s) (where available) was maintained between day 0 and after 4 weeks of culture (Figure 4a and Supplementary Table S4). Furthermore, although the clonality was not altered, the cell fold expansion observed was substantial (ranging from 50 to more than 600 times), now making it feasible to use multiple approaches/techniques usually limited by the small number of available cells (Figure 5a and Supplementary Table S5). It is worth noting that MSCs recovered from RAEB patients were nonsupportive as indicated by the low hCD45^+^ cell expansion. Therefore, we decided to determine whether murine stroma cell line MS5 could be used as an alternative to hMSC stromal support for RAEB cases where we observed poor recovery of hMSCs, as has also been previously reported.^14^ We observed a substantial increase in the expansion of CD34^+^ cells from RAEB patients. Notably, patient CD34^+^ cells grown on mouse stroma now exhibited a similar fold expansion as was observed in MSC stroma for RARS as well as RCMD patients (Figure 5a).

Next, we investigated the potential of LTC expanded MDS cells to form long-term culture-initiating cells; bearing in mind that the impressive cell fold expansion observed on stroma could have caused an exhaustion of the more primitive malignant cells (Figure 5a). Both MDS culture on MSCs and on MS5 were able to give rise to colonies (Figure 5b) with a ratio of burst forming unit- erythroid/colony forming unit-granulocyte-macrophage (BFU-E/CFU-GM) colonies similar to that observed at day 0 (Figure 5c). Finally, we wanted to determine whether 4 weeks of expansion on a monolayer of stroma cells would have induced any additional chromosomal instability within the MDS patient cells. Therefore, we performed single-nucleotide polymorphism karyotyping (Affymetrix GeneChip System) on pre- and post-LTC-ICs for 3 patients. Our data showed that the clones were stable during the co-culture and did not induce any additional chromosomal abnormalities. Importantly, in one patient (MDS48) a minor clone (<25%) carrying trisomy-8 was also maintained in hCD45^+^ cells recovered from the MS5 stroma conditions (Figure 4b and Supplementary Figure S5). Our data clearly demonstrates that this 2D *in vitro* system can be used with a small number of CD34^+^ cells (often observed) as a surrogate model to study the therapeutic strategies as well as the potential mechanisms of drug resistance observed often in MDS patients.

In this report, we used MNCs or CD34^+^ primary MDS cells and autologous/allogeneic hMSCs injected intra-BM into different immunodeficient mouse models. Our results showed that although it is possible to xenotransplant MDS patient cells, the engraftment remains low, with or without the coinjection of MSCs, therefore compromising the test of new therapeutic strategies *in vivo*. We showed that human MSCs do not last more than a week in the mouse BM, therefore suggesting that other transplantation methods like the use of a three-dimensional scaffold that others and we have recently reported using acute myeloid leukemia primary samples^15, 16^ could be of potential use to better dissect the role if any of MSCs in supporting the MDS clones. Although the development of preclinical *in vivo* models is necessary, we have demonstrated the potential value of the 2D co-culture system using MSCs (or murine MS5) as an alternative model to study MDS. This *ex vivo* culture system, which lasts for only 4 weeks and requires low number of human CD34^+^ cells, provides a robust preclinical assessment model to test therapeutic effects of different drugs and other approaches on the MDS clonality before treatment of MDS patients as well as provides a model to better dissect the cross-talk between MSCs and the malignant clones.

## Figures and Tables

**Table 1:** Clinical data for MDS patients used in this study

*UPN*	*Sex*	*WHO diagnosis*	*Blasts (%)*	*Cytogenetics*
MDS1	M	RCMD	3	47,XY,+8 [20]
MDS2	M	RCMD	3	45,XY-7[8]/46,XY[2]
MDS3	F	RAEBII	17	46,XX,del(7)(q21q36) [28]/46,XX [2]
MDS4	M	RCMD-RS	2	47,XY,del(20)(q11q13),+21[30]
MDS5	M	RARS	2	46,XY [20]
MDS6	M	MDS/MPN	8	47,XY,+8 [30]
MDS7	M	RCMD	0	46,XY [20]
MDS9	M	RARS-T	1	46,XY [20]
MDS13	M	RCMD	1	46,XY[7]
MDS14	F	RCMD	2	46,XX,del(5)(q13q31),del(20)(q11q13) [20] /46,XX [10]
MDS17	M	RAEBII	11	46,XY,-7,+mar[10]
MDS20	F	RCMD-RS	1	46,XX [20]
MDS22	M	RCMD	2	46,XY [20]
MDS24	M	RAEBI	9	46,XY[20]
MDS25	M	RARS	0	46,XY [20]
MDS27	M	RCMD-RS	3	46,XY [20]
MDS35	F	Del5q	2	46,XX,del(5)(q13q31) [27] / 46,XX [3]
MDS36	M	RCMD	0	46,XY [20]
MDS37	M	RCMD	1	46,XY [20]
MDS38	M	RCMD	3	46,XY [20]
MDS39	F	RCMD	2	46,XX[20]
MDS40	M	RCMD	2	46,XY [20]
MDS41	M	RCMD	2	46,XY [20]
MDS42	F	RCMD	3	46,XX [10]
MDS43	M	RAEBII	11	46,XY [20]
MDS44	F	RAEBII	15	46,XX [20]
MDS45	M	RCMD	3	46,XY[20]
MDS46	M	RCMD	2	46,XY [20]
MDS47	M	RARS	0	46,XY [20]
MDS48	M	RARS	3	47,XY,+8[10]/46,XY[10]
MDS49	F	RARS	1	46,XX [20]
MDS50	F	RAEBII	2.8	48 XX, 20q-, Trisomy-8, Trisomy-11
MDS51	M	CMML	1.7	46,XY [20]
MDS52	F	RARS	0.1	46 X,i(X)(q13) [10], 46 XX [12]
MDS53	M	CMML	0.7	46 XY del 20 q12 [9], 46 XY [3]
MDS54	F	RAEBI	7.4	46 XX
MDS55	F	RAEBII	8.8	-4, -7 der5, -13, -5
MDS56	M	CMML	3.5	46 XY [23]

Abbreviations: CMML, chronic myelomonocytic leukemia; F, female; M, male; MDS, myelodysplastic syndrome; MDS/MPN, myelodysplastic/myeloproliferative neoplasm; RAEB, refractory anemia with excess blasts; RARS, refractory anemia with ringed sideroblasts; RARS-T, refractory anemia with ring sideroblasts and thrombocytosis; RCMD, refractory cytopenia with multilineage dysplasia; RCMD-RS, refractory cytopenia with multilineage dysplasia and ringed sideroblasts; WHO, World Health Organization.
