# Supplementary material for: Preclinical modeling of myelodysplastic syndromes
Source: Leukemia. 2017 Jun 30;31(12):2702–8. doi: 10.1038/leu.2017.172 (PMC5729336; doi:10.1038/leu.2017.172)
Supplement: Supplementary Material [file leu2017172x1.docx]

**Supplementary**

**Supplementary methods**

**MNC, CD34^+^ and MSC Isolation from bone marrow**

Mononuclear cells (MNCs) were isolated from the bone marrow cells by centrifugation using Ficoll-Paque^TM^ PLUS (GE Healthcare Life Sciences, Buckinghamshire, UK). CD34^+^ cell enrichment was performed using an Easysep Human CD34 positive selection kit (cat no 18056) and Easysep magnet (StemCell Technologies, Vancouver, Canada) according to the manufacturer's instructions. Isolated CD34^-^ fraction during the CD34^+^ cell selection was used for Mesenchymal Stroma Cells (MSCs) isolation and followed by expansion.

**MSC Expansion**

CD34^-^ cells were seeded at a concentration of 1.10^6^/cm^2^, in Alpha MEM low glucose (Life Technologies, Paisley, UK) supplemented with FBS (Fetal Bovine Serum, Mesenchymal Stem Cell Qualified, life technologies, Paisley, UK). Culture media was replaced 24hours after plating, and cells were frozen at passage 2.

**Bioluminescence imaging**

Isofluorane-anesthetized animals were imaged using the Xenogen IVIS imaging system 20-55 min after D-luciferin (Caliper Life Sciences, Cambridge, UK) was injected intra-peritoneally (150mg/kg). Bioluminescence images were taken from ventral side of the mice. The photons emitted from luciferase-expressing MSC cells, expressed as Flux (photons/second/cm^2^/steradian) were quantified and analyzed using the ‘Living image’ software (Caliper Life Science).

**Immunofluorescence of Mouse Bones**

Harvested bones were fixed overnight in 10% neutral buffered formalin and then decalcified with 17% EDTA (Osteosoft, Millipore) for 7 days. Following on, bones were processed, paraffin embedded and sectioned (5 μm) for histological studies. Hematoxylin/eosin was performed first to assess quality of the sections. For immunofluorescence (IF) studies heat antigen retrieval was performed in all cases. Primary unconjugated antibodies employed were specific for the following proteins: human CD45 (Dako, M0701), Endomucin (Santa Cruz, sc-65495) and GFP^+^ cells (Santa Cruz, sc-8334). Secondary fluorescent antibodies were from Invitrogen. Images were obtained using Zeiss Axio Scan.Z1 slice scanner and with Zen blue edition software. Data from images was obtained using Fiji software equipped with both grid overlay and Cell Counter plugins.

**Whole-exome sequencing and data analysis**

qDNA (non-WGA, 100-500ng) was processed for whole-exome sequencing (Agilent V4) and sequenced on the Illumina HiSeq2000 or HiSeq2500 (Paired end V3 chemistry) according to manufacturer’s instructions. Base calling was generated by the Illumina RTA software. Demultiplexing and conversion of basecalls to fastq was performed by Casava version 1.8.2, filtering out poor quality reads fastq files concatenated. Alignment, realignment, recalibration as well as downstream data analysis was performed as described previously^1^.

**AML Myeloid gene panel**

Gene mutation screening for myeloid-related genes was performed by using King’s College Hospital myeloid-gene panel. gDNA from patient bone marrow total nucleated cells was used to amplify entire coding regions or known hotspots: ASXL1, CBL, CEBPA, DNMT3A, ETV6, EZH2, FLT3, GATA2, IDH1, IDH2, JAK2, KDM6A, KIT, KRAS, NPM1, NRAS, RUNX1, SF3B1, SRSF2, STAG2, TET2, TP53, U2AF1 and ZRSR2; using the Illumina TruSeq Amplicon panel as described previously^2^. Sequencing was performed using the MiSeq Instrument with version 3 sequencing chemistry (Illumina, San Diego, CA, USA), as per the manufacture's protocol. Following on, sequencing data was processed using the GATK pipeline package (Broad Institute, Boston, MA, USA). Processed VCF and BAM files were visualized using variant studio (Illimina) and integrated genome viewer (IGV), respectively. Candidate variants obtained were passed for validation if the variants were deemed to cause protein changes and not found in dbSNP142, esp5400 and 6500, and 1000 genomes databases at <0.01 population allele frequency.

Gene variants were deemed to be candidate variants and were included for further analysis only if they had an allele burden of ≥5%, present in COSMIC (Catalogue of Somatic Mutations in Cancer) or had been previously reported. In addition, novel variants were also included if the coverage across the variant was >200 sequencing reads. All the candidate variants were subjected to confirmation via independent targeted PCR followed by transposon-based Illumina Nextera technology sequencing as described in next section.

**Sequencing validation Experiments**

PCR amplicon libraries were prepared as described previously (Mian et al 2015). DNA from human bone marrow total nucleated cells (Day 0), hCD33^+^ post-xenografted mice and/or post-LTC hCD45^+^ was used for PCR library preparation. After the PCR amplification of the targeted gene regions, transposon-based Illumina Nextera technology (Illumina, UK) was used to prepare the Illumina sequencing libraries by following the manufacturer’s protocol. All patient indexed amplicon mixes were quantified using the Quant-iT pico-green dsDNA assay kit (Life Science Technologies) by following manufacturers protocol. Subsequently, amplified patient libraries were pooled together, purified using the Agencourt AMpure XP beads (Beckman Coulter). Libraries were quantitated using the Quant-iT picoGreen dsDNA assay kit (Life Science Technologies) by following manufacturers protocol. Pooled libraries were sequenced on the Illumina MiSeq platform utilizing version 2 chemistry with 150-250 paired-end reads. On average gene amplicons were covered with >1000 sequencing reads, therefore providing a high depth mutation screening.

**Affy SNP Karyotyping**

Genomic DNA was extracted using the Blood Mini DNA Extraction Kit (Qiagen, Manchester, UK) and processed for SNP array analysis using the Affymetrix CytoHD (Affymetrix, Woodburn Green, UK), all as per the manufacturer’s protocol. SNP array data was quality checked by the following parameters; Waviness SD<0.12, SNPQC>15 and MAPD<0.25.

Array data not satisfying these criteria were visually inspected to determine quality and repeated if necessary. Affymetrix CEL files were processed using Affymetrix Genotyping Console 3.0 and analysed using Chromosome Analysis Suite (ChAS v2.0, Affymetrix). CN aberrations having >50% overlap with variants present in the database of genomic variants and a reference collated from 1000 healthy subjects (Affymetrix) were excluded from further analysis. Regions of CN-LOH were excluded if they were <20 Mb and located interstitial in the chromosome. Regions of CN-LOH that included the telomeric end were included for analysis, irrespective of size.

**Statistical Analysis**

Statistical analysis was performed with Prism Version 6 software (GraphPad). Statistical analysis was performed using the two-tailed student’s t-test and ANOVA to determine the level of significance.

**Supplementary Figure 1:** Distribution of hCD45+ engraftment in total mouse bone marrow at sacrifice from NSG mice injected with CD34+ cells from MDS patients (patients n= 22; NSG mice n= 74). hCD45 ≥ 0.01% in total mouse bone marrow was considered as successful human cell engraftment.

**Supplementary Figure 2:** Representative **i**mmunofluorescence images showing the presence or absence of GFP^+^ cells (red) in injected femur bone from NSG or NSG-S mice after 12 weeks. hMSCs-Red, hCD45^+^-Green, Dapi-Blue. Panel 6 and 7 represent femur bone from the two mice that had GFP+ cells.

**
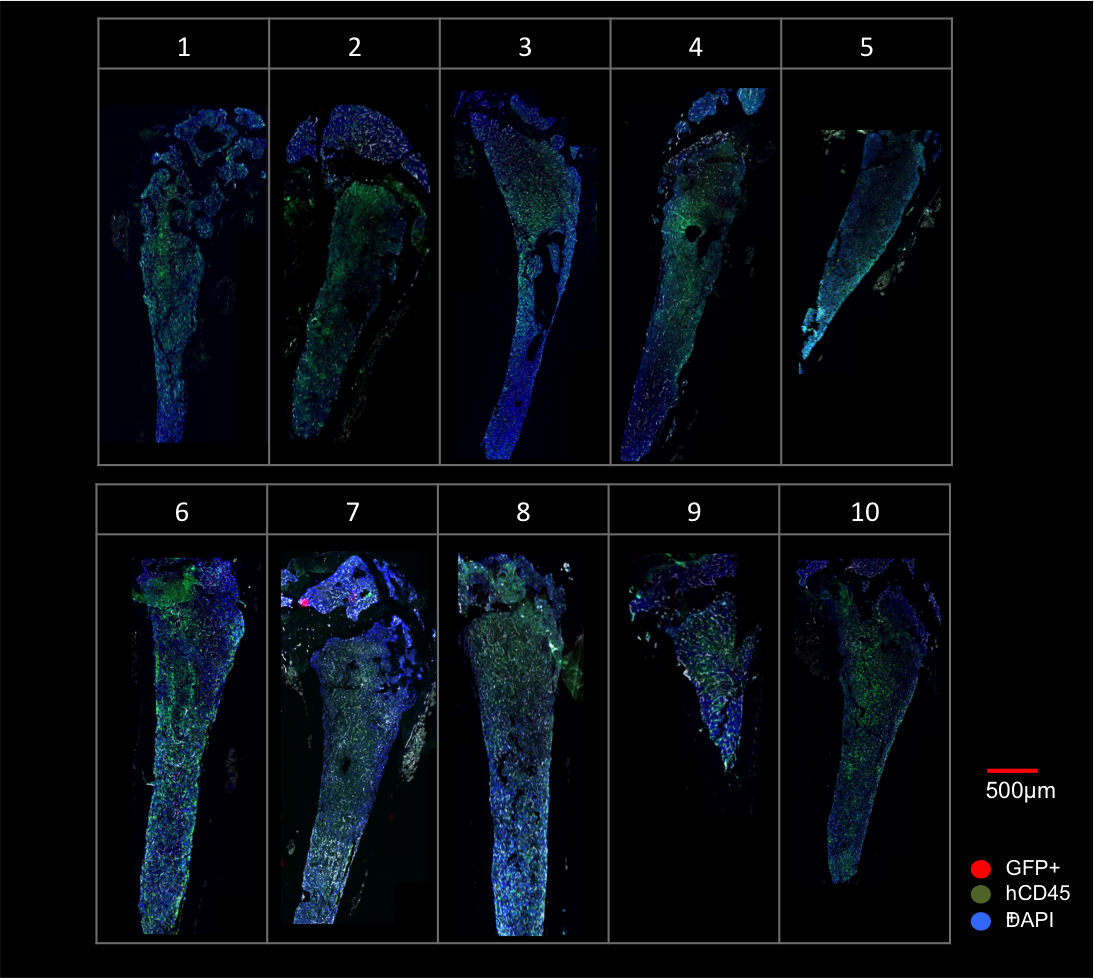
**

**Supplementary Figure 3:** Representative immunofluorescence images showing the presence (injected Femur, left image and bone 7) or absence (right femur 6 and articulated tibia) of GFP+ cells (red) at 12 weeks. hMSCs-Red, hCD45+-Green, Dapi-Blue.

**
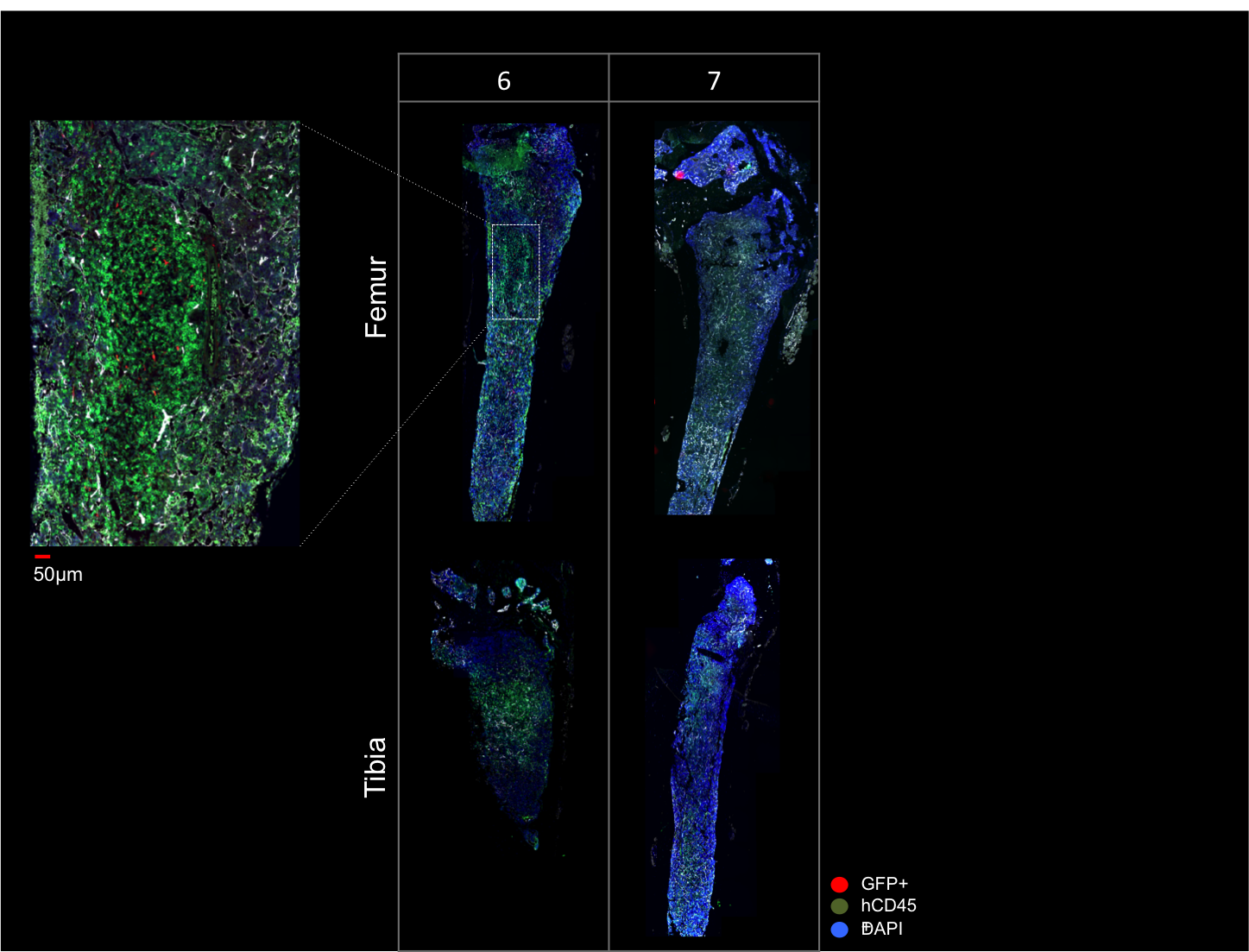
**

**Supplementary Figure 4: 2D *In Vitro* modeling of MDS.**

(A) Schematic representation of isolation of hCD34^+^ cells and MSCs from MDS patient bone marrow used for colony-forming assay, long-term culture and sequencing analysis.

(B) Representative pictures of hCD45^+^ cells and their stroma (MSCs vs MS5) at 4 weeks from two patients.

**Supplementary Figure 5:** SNP karyotyping showing the maintenance of MDS clonality between pre- and post-culture for two patients.

**
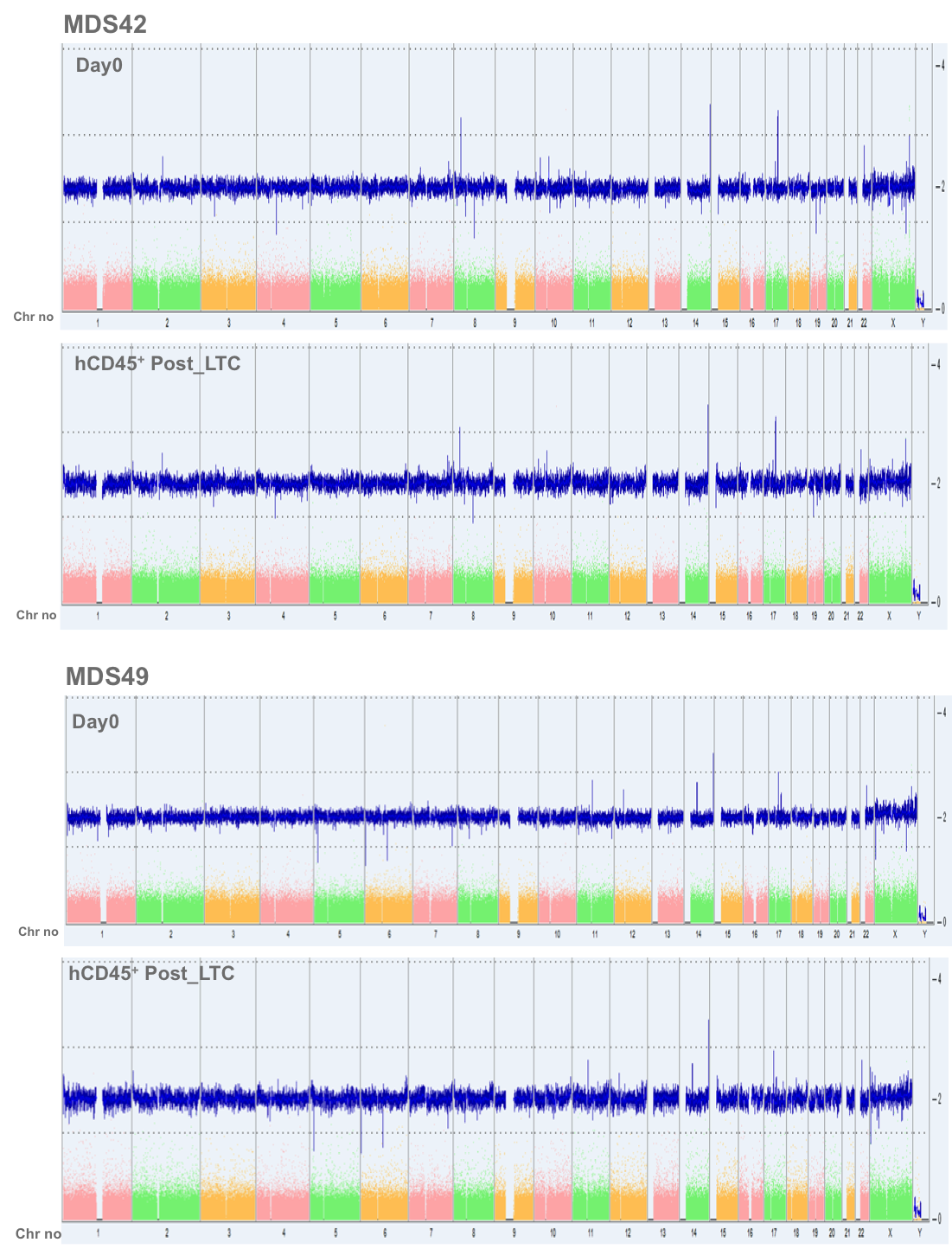
**

**Supplementary Table 1:** Somatic mutations detected in MDS patients using whole-exome sequencing or Targeted AML myeloid-gene Panel. * Whole-exome sequencing.

| UPN | Chr | Start | End | Ref | Alt | Mutation Type | Gene Name | Amino Acid Change | MAB |
| --- | --- | --- | --- | --- | --- | --- | --- | --- | --- |
| MDS1* | chrX | 15836766 | 15836766 | G | A | splicing | ZRSR2 | SS 827+1G>A | 83% |
| MDS1* | chr4 | 106180857 | 106180857 | C | A | nonsense | TET2 | Y1295X | 47% |
| MDS1* | chr10 | 73050842 | 73050842 | G | A | missense | UNC5B | V424I | 48% |
| MDS1* | chr22 | 26849216 | 26849216 | C | T | missense | HPS4 | G704R | 43% |
| MDS1* | chr11 | 108218026 | 108218026 | G | T | missense | ATM | G2869C | 46% |
| MDS1* | chr8 | 53586699 | 53586702 | TCTT | - | nonsense | RB1CC1 | K235fs | 37% |
| MDS2* | chr20 | 31021211 | 31021211 | C | T | nonsence | ASXL1 | R404X | 26% |
| MDS2* | chr21 | 44514777 | 44514777 | T | G | missence | U2AF1 | Q84P | 24% |
| MDS2* | chr6 | 43023677 | 43023677 | C | T | missence | MRPL2 | E197K | 29% |
| MDS2* | chr6 | 33371285 | 33371285 | C | T | missence | KIFC1 | P110S | 29% |
| MDS3* | chr2 | 25457242 | 25457242 | C | T | missence | DNMT3A | R882H | 51% |
| MDS3* | chr2 | 209113113 | 209113113 | G | A | missence | IDH1 | R132C | 52% |
| MDS3* | chr13 | 103386993 | 103386993 | C | T | missence | CCDC168 | D5352N | 59% |
| MDS3* | chr9 | 5073770 | 5073770 | G | T | missence | JAK2 | V617F | <1% |
| MDS4 | chr17 | 74732959 | 74732959 | G | A | missense | SRSF2 | P95L | 24% |
| MDS4 | chr21 | 36252945 | 36252945 | G | C | missense | RUNX1 | N139L | 40% |
| MDS5* | chr2 | 198267371 | 198267371 | G | C | missence | SF3B1 | H662Q | 42% |
| MDS5* | chr5 | 151175137 | 151175137 | G | C | splicing | G3BP1 | SS Exon 6 G>C | 40% |
| MDS5* | chr2 | 25467133 | 25467133 | C | G | missence | DNMT3A | W581S | 41% |
| MDS5* | chr10 | 98145885 | 98145885 | A | G | missence | TLL2 | V647A | 7% |
| MDS5* | chr1 | 183230405 | 183230405 | C | T | missence | NMNAT2 | V224M | 41% |
| MDS5* | chr22 | 42463872 | 42463872 | T | A | missence | NAGA | Y74F | 35% |
| MDS5* | chr2 | 169833167 | 169833167 | C | T | missence | ABCB11 | G410S | 38% |
| MDS5* | chr1 | 27238545 | 27238545 | C | T | missence | NR0B2 | G189R | 29% |
| MDS5* | chr13 | 24797870 | 24797870 | G | A | missence | SPATA13 | R268Q | 41.00% |
| MDS5* | chr1 | 46088498 | 46088498 | A | G | missence | CCDC17 | L213P | 17.00% |
| MDS6* | chr7 | 148526910 | 148526910 | G | T | missence | EZH2 | P132T | 97% |
| MDS6* | chr21 | 36171653 | 36171653 | - | A | nonsence | RUNX1 | P304fs | 44% |
| MDS6* | chr20 | 31022439 | 31022443 | GGAGG | - | nonsence | ASXL1 | G642fs | 18% |
| MDS6* | chr1 | 241901671 | 241901671 | C | T | missence | WDR64 | R391W | 51% |
| MDS6* | chr11 | 101775568 | 101775568 | G | A | missence | ANGPTL5 | P139L | 54% |
| MDS6* | chr20 | 58330363 | 58330363 | C | T | missence | PHACTR3 | T121M | 49% |
| MDS7 | chr20 | 31022402 | 31022402 | CACCACTGCCATAGAGAGGCGGC | - | nonsense | ASXL1 | E635RfsX15 | 15.00% |
| MDS7 | chr21 | 36252874 | 36252874 | A | T | missense | RUNX1 | F163Y | 8.61% |
| MDS7 | chr21 | 44514777 | 44514777 | T | G | missense | U2AF1 | Q157P | 22.60% |
| MDS8 | None Detected | - | - | - | - | - | - | - | - |
| MDS9* | chr2 | 198266834 | 198266834 | T | C | missence | SF3B1 | K700E | 43% |
| MDS9* | chr9 | 5073770 | 5073770 | G | T | missence | JAK2 | V617F | 36% |
| MDS9* | chr2 | 25470588 | 25470588 | C | G | missence | DNMT3A | V107L | 4% |
| MDS9* | chr1 | 16091611 | 16091611 | G | A | missence | FBLIM1 | A45T | 27% |
| MDS9* | chr6 | 26124820 | 26124820 | G | T | missence | HIS1H2AC | K120N | 31% |
| MDS9* | chr10 | 118434314 | 118434314 | C | T | missence | HSPA12A | G669E | 2.00% |
| MDS9* | chr3 | 108475355 | 108475355 | C | T | missence | RETNLB | G70R | 28.00% |
| MDS9* | chr2 | 17897511 | 17897511 | T | A | missence | SMC6 | K456M | 2.00% |
| MDS9* | chr2 | 174777831 | 174777831 | C | T | missence | SP3 | D598N | 4.00% |
| MDS9* | chr11 | 2189733 | 2189733 | C | T | missence | TH | A159T | 15.00% |
| MDS13* | chr15 | 90631934 | 90631934 | C | T | missence | IDH2 | R140Q | 50.60% |
| MDS13* | chr8 | 35624475 | 35624475 | T | C | missence | UNC5D | F790S | 44.86% |
| MDS14 | chr21 | 36171729 | 36171729 | c | t | nonsence | RUNX1 | W279X | 32.00% |
| MDS17* | chr21 | 44514777 | 44514777 | T | C | missence | U2AF1 | Q84R | 52.40% |
| MDS17* | chr9 | 5073770 | 5073770 | G | T | missence | JAK2 | V617F | 41.67% |
| MDS17* | chr4 | 106190819 | 106190819 | G | T | missence | TET2 | R1366L | 5.49% |
| MDS17* | chr4 | 106155779 | 106155779 | A | - | nonsence | TET2 | E227fs | 50.75% |
| MDS17* | chr9 | 126132408 | 126132408 | T | C | missence | CRB2 | V359A | 33.00% |
| MDS17* | chr12 | 80887102 | 80887102 | A | G | missence | PTPRQ | M298V | 52.00% |
| MDS17* | chr17 | 7578475 | 7578475 | - | G | nonsense | TP53 | P113fs | 5.00% |
| MDS18 | chr21 | 44524456 | 44524456 | G | A | missense | U2AF1 | S34F | 27.72% |
| MDS20* | chr2 | 198266834 | 198266834 | T | C | missense | SF3B1 | K700E | 40.00% |
| MDS20* | chr19 | 50728875 | 50728875 | C | G | missense | MYH14 | P251A | 44.00% |
| MDS24 | None Detected | - | - | - | - | - | - | - | - |
| MDS25* | None Detected |  |  |  |  |  |  |  |  |
| MDS27* | chr2 | 198267371 | 198267371 | G | T | missence | SF3B1 | H662Q | 28% |
| MDS27* | chr6 | 84108234 | 84108234 | A | G | missence | ME1 | Y72H | 31% |
| MDS27* | chr1 | 7731086 | 7731086 | G | T | missence | CAMTA1 | C923F | 33% |
| MDS27* | chr9 | 96860488 | 96860488 | A | T | missence | PTPDC1 | A1640T | 21% |
| MDS35 | chr15 | 90631934 | 90631934 | C | T | missence | IDH2 | R140Q | 41% |
| MDS36 | chr17 | 74732959 | 74732959 | G | T | missence | SRSF2 | P95H | 36.96 |
| MDS37* | chr16 | 56448181 | 56448181 | - | TT | nonsense | AMFR | R111fs | 36% |
| MDS37* | chr2 | 127808820 | 127808820 | C | T | splicing | BIN1 | SS 1372-1G>A | 11% |
| MDS37* | chr7 | 101839974 | 101839974 | C | - | nonsense | CUX1 | A428fs | 16% |
| MDS37* | chr2 | 25461998 | 25461998 | G | T | splicing | DNMT3A | SS 2408+1G>T | 5% |
| MDS37* | chr14 | 102495991 | 102495991 | A | T | missence | DYNC1H1 | E3195V | 26% |
| MDS37* | chr11 | 92577541 | 92577541 | C | T | missence | FAT3 | R3670W | 11% |
| MDS37* | chr4 | 153893616 | 153893616 | G | T | missence | FHDC1 | D436Y | 9% |
| MDS37* | chr9 | 5073770 | 5073770 | G | T | missence | JAK2 | V617F | 4% |
| MDS37* | chr12 | 58162891 | 58162891 | T | A | missence | METTL1 | E240V | 27% |
| MDS37* | chr6 | 79724825 | 79724827 | CTC | - | nonsense | PHIP | 499_500del | 29% |
| MDS37* | chr17 | 6406885 | 6406885 | G | T | missence | PITPNM3 | T79N | 37% |
| MDS37* | chr4 | 3318579 | 3318579 | G | A | missence | RGS12 | V228M | 16% |
| MDS37* | chr21 | 36206709 | 36206709 | - | GC | nonsense | RUNX1 | Q268fs | 21% |
| MDS37* | chr2 | 198266834 | 198266834 | T | C | missence | SF3B1 | K700E | 39% |
| MDS37* | chr4 | 106158220 | 106158220 | T | - | nonsense | TET2 | F1041fs | 43% |
| MDS37* | chr11 | 134118366 | 134118366 | A | G | missence | THYN1 | F215S | 32% |
| MDS37* | chr4 | 153690836 | 153690836 | T | A | missence | TIGD4 | T441S | 12% |
| MDS37* | chr17 | 7577569 | 7577569 | A | G | missence | TP53 | C199R | 2% |
| MDS37* | chr15 | 31295053 | 31295053 | T | - | nonsense | TRPM1 | S1284fs | 52% |
| MDS37* | chr12 | 863422 | 863422 | T | G | missence | WNK1 | S231A | 47% |
| MDS38* | chr1 | 33138418 | 33138418 | G | T | missence | RBBP4 | K376N | 34.69% |
| MDS38* | chr1 | 45115622 | 45115622 | G | A | missence | RNF220 | R541Q | 25.45% |
| MDS38* | chr10 | 102262242 | 102262242 | G | A | splicing | SEC31B | SS 1180-1G>A | 32.26% |
| MDS38* | chr4 | 106164764 | 106164764 | G | A | missence | TET2 | C1211Y | 25.75% |
| MDS38* | chrX | 47069087 | 47069087 | - | T | splicing | UBA1 | SS 2003+1->T | 70.09% |
| MDS38* | chrX | 119387672 | 119387672 | - | A | nonsense | ZBTB33 | N134fs | 15.91% |
| MDS38* | chr6 | 43307691 | 43307691 | G | A | nonsense | ZNF318 | R1349X | 35.51% |
| MDS38* | chr16 | 20693591 | 20693591 | G | A | nonsense | ACSM1 | R200X | 38.71% |
| MDS38* | chr12 | 53291346 | 53291346 | C | T | missence | KRT8 | G440S | 38.10% |
| MDS38* | chr2 | 198266834 | 198266834 | T | C | missence | SF3B1 | K700E | 43.75% |
| MDS38* | chr17 | 7579321 | 7579321 | - | A | nonsense | TP53 | V83fs | 6.27% |
| MDS38* | chr8 | 77775639 | 77775639 | G | A | missence | ZFHX4 | G3230D | 44.52% |
| MDS39* | None Detected | - | - | - | - | - | - | - | - |
| MDS40 | None Detected | - | - | - | - | - | - | - | - |
| MDS41 | None Detected | - | - | - | - | - | - | - | - |
| MDS42 | None Detected | - | - | - | - | - | - | - | - |
| MDS43 | chr20 | 31022983 | 31022983 | T | G | nonsence | ASXL1 | Leu823X | 40% |
| MDS43 | chr21 | 36164854 |  | C | - | nonsense | RUNX1 | S341PfsX253 | 40% |
| MDS44* | chr3 | 179294422 | 179294422 | C | T | missence | ACTL6A | P154S | 38% |
| MDS44* | chr4 | 100260753 | 100260753 | G | A |  | ADH1C | - | 98% |
| MDS44* | chr22 | 29121242 | 29121242 | G | A | missence | CHEK2 | R188W | 51% |
| MDS44* | chrX | 153588236 | 153588236 | G | C | missence | FLNA | D1281E | 37% |
| MDS44* | chr5 | 121188065 | 121188065 | G | A | missence | FTMT | R136Q | 45% |
| MDS44* | chr3 | 185375122 | 185375122 | C | A | missence | IGF2BP2 | K446N | 48% |
| MDS44* | chr21 | 31971022 | 31971022 | A | T | missence | KRTAP6-2 | S58T | 42% |
| MDS44* | chr1 | 43818193 | 43818193 | A | T | missence | MPL | K553M | 44% |
| MDS44* | chr12 | 110032875 | 110032875 | G | A | missence | MVK | V310M | 48% |
| MDS44* | chr13 | 79908507 | 79908507 | - | T | nonsense | RBM26 | T933fs | 50% |
| MDS44* | chr21 | 36253001 | 36253002 | GG | - | nonsense | RUNX1 | A120fs | 30% |
| MDS44* | chr8 | 70744087 | 70744087 | A | C | missence | SLCO5A1 | I274M | 46% |
| MDS44* | chr17 | 74732936 | 74732959 | GGCGGCTGTGGTGTGAGTCCGGGG | - | nonsense | SRSF2 | 95_103del | 38% |
| MDS44* | chr14 | 51448722 | 51448722 | A | G | missence | TRIM9 | V568A | 47% |
| MDS45* | chr8 | 41753983 | 41753983 | T | A | nonsense | ANK1 | K6X | 27% |
| MDS45* | chr2 | 220404776 | 220404776 | G | A | nonsense | CHPF | Q553X | 41% |
| MDS45* | chr6 | 75798872 | 75798872 | C | T | missence | COL12A1 | G2987D | 43% |
| MDS45* | chr10 | 96447978 | 96447978 | A | G | missence | CYP2C18 | D143G | 38% |
| MDS45* | chr10 | 85984219 | 85984219 | C | G | missence | LRIT2 | Q254H | 31% |
| MDS45* | chr12 | 133212563 | 133212563 | C | T | missence | POLE | R1909Q | 30% |
| MDS45* | chr4 | 106197285 | 106197285 | T | C | missence | TET2 | I1873T | 32% |
| MDS45* | chr4 | 106164061 | 106164061 | C | T | nonsense | TET2 | Q1191X | 39% |
| MDS45* | chrX | 15833938 | 15833938 | - | T | nonsense | ZRSR2 | T232fs | 48% |
| MDS46* | chr5 | 178772220 | 178772220 | G | A | missence | ADAMTS2 | A37V | 47% |
| MDS46* | chr19 | 18979615 | 18979615 | - | G | nonsense | GDF1 | V304fs | 59% |
| MDS47* | chr2 | 198266834 | 198266834 | T | C | nonsense | SF3B1 | K700E | 44% |
| MDS47* | chr8 | 77775639 | 77775639 | G | A | nonsense | ZFHX4 | G3230D | 46% |
| MDS47* | chr12 | 53291346 | 53291346 | C | T | nonsense | KRT8 | G440S | 38% |
| MDS47* | chr16 | 20693591 | 20693591 | G | A | nonsense | ACSM1 | R200X | 39% |
| MDS47* | chr17 | 7579321 | 7579321 | - | A | nonsense | TP53 | V83fs | 6% |
| MDS48* | chr2 | 24086364 | 24086364 | T | C | missence | ATAD2B | N456D | 40% |
| MDS48* | chr2 | 242593026 | 242593026 | G | A | splicing | ATG4B | SS 283+1G>A | 44% |
| MDS48* | chr7 | 40118317 | 40118317 | A | G | splicing | CDK13 | SS 2898-2A>G | 10% |
| MDS48* | chr1 | 230827277 | 230827277 | G | T | missence | COG2 | Q694H | 9% |
| MDS48* | chr2 | 25463289 | 25463289 | T | G | missence | DNMT3A | Y735S | 49% |
| MDS48* | chr6 | 46107688 | 46107688 | A | G | missence | ENPP4 | N123S | 11% |
| MDS48* | chr6 | 28251732 | 28251732 | C | T | missence | PGBD1 | R48W | 33% |
| MDS48* | chr2 | 1647328 | 1647328 | C | T | missence | PXDN | G1255E | 41% |
| MDS48* | chr2 | 198266834 | 198266834 | T | C | missence | SF3B1 | K700E | 45% |
| MDS48* | chr4 | 106157711 | 106157711 | - | TG | nonsense | TET2 | N871fs | 3% |
| MDS48* | chr4 | 106182957 | 106182957 | - | AA | nonsense | TET2 | L1332fs | 7% |
| MDS48* | chr4 | 106164793 | 106164793 | T | C | missence | TET2 | C1221R | 10% |
| MDS48* | chr4 | 106196374 | 106196374 | C | A | nonsense | TET2 | Y1569X | 42% |
| MDS48* | chr17 | 3990782 | 3990782 | A | - | nonsense | ZZEF1 | F763fs | 43% |
| MDS49* | chr2 | 25457192 | 25457192 | G | A | missence | DNMT3A | R899C | 6% |
| MDS49* | chr2 | 25470002 | 25470002 | A | G | missence | DNMT3A | L347P | 35% |
| MDS49* | chr4 | 185340632 | 185340632 | T | C | missence | IRF2 | I60V | 45% |
| MDS49* | chr2 | 198267491 | 198267491 | C | G | missence | SF3B1 | E622D | 30% |
| MDS49* | chr4 | 106193892 | 106193892 | C | T | nonsence | TET2 | R1452X | 6% |

**Supplementary Table 2:** Somatic mutations detected at day 0 and following Xenotransplantation.

| UPN | Gene | Amino Acid Change | Day0 MAB | NSG Mouse1 MAB | NSG Mouse2 MAB | NSG Mouse3 MAB | NSGS Mouse1 MAB | NSGS Mouse2 MAB | NSGS Mouse3 MAB | NSGS Mouse3 MAB2 |
| --- | --- | --- | --- | --- | --- | --- | --- | --- | --- | --- |
| MDS1 | ZRSR2 | SS 827+1G>A | 83% | 7% |  |  |  |  |  |  |
| MDS1 | TET2 | Y1295X | 47% | 13% |  |  |  |  |  |  |
| MDS1 | UNC5B | V424I | 48% | 8% |  |  |  |  |  |  |
| MDS1 | HPS4 | G704R | 43% | 9% |  |  |  |  |  |  |
| MDS1 | ATM | G2869C | 46% | 9% |  |  |  |  |  |  |
| MDS1 | RB1CC1 | K235fs | 37% | 9% |  |  |  |  |  |  |
| MDS3 | DNMT3A | R882H | 51% | 37% |  |  |  |  |  |  |
| MDS3 | IDH1 | R132C | 52% | 53% |  |  |  |  |  |  |
| MDS3 | CCDC168 | D5352N | 59% | 56% |  |  |  |  |  |  |
| MDS3 | JAK2 | V617F | <1% |  |  |  |  |  |  |  |
| MDS5 | SF3B1 | H662Q | 42% | 38% |  |  |  |  |  |  |
| MDS5 | G3BP1 | SS Exon 6 G>C | 40% | 45% |  |  |  |  |  |  |
| MDS5 | DNMT3A | W581S | 41% | 39% |  |  |  |  |  |  |
| MDS5 | TLL2 | V647A | 7% | 1% |  |  |  |  |  |  |
| MDS5 | NMNAT2 | V224M | 41% | 40% |  |  |  |  |  |  |
| MDS5 | NAGA | Y74F | 35% | 44% |  |  |  |  |  |  |
| MDS5 | ABCB11 | G410S | 38% | 32% |  |  |  |  |  |  |
| MDS5 | NR0B2 | G189R | 29% | 31% |  |  |  |  |  |  |
| MDS5 | SPATA13 | R268Q | 41% | 44% |  |  |  |  |  |  |
| MDS5 | CCDC17 | L213P | 17% | 17% |  |  |  |  |  |  |
| MDS6 | EZH2 | P132T | 97% | 99% |  |  |  |  |  |  |
| MDS6 | RUNX1 | P304fs | 44% | 51% |  |  |  |  |  |  |
| MDS6 | ASXL1 | G642fs | 18% | 23% |  |  |  |  |  |  |
| MDS6 | WDR64 | R391W | 51% | 50% |  |  |  |  |  |  |
| MDS6 | ANGPTL5 | P139L | 54% | 47% |  |  |  |  |  |  |
| MDS6 | PHACTR3 | T121M | 49% | 40% |  |  |  |  |  |  |
| MDS9 | SF3B1 | K700E | 43% | 46% | 46% | 50% |  |  |  |  |
| MDS9 | JAK2 | V617F | 36% | 17% | 10% | 17% |  |  |  |  |
| MDS9 | DNMT3A | V107L | 4% | 10% | 9% | 12% |  |  |  |  |
| MDS9 | FBLIM1 | A45T | 27% | 11% | 6% | 8% |  |  |  |  |
| MDS9 | HIS1H2AC | K120N | 31% | 14% | 10% | 14% |  |  |  |  |
| MDS9 | HSPA12A | G669E | 2% | 7% | 7% | 6% |  |  |  |  |
| MDS9 | RETNLB | G70R | 28% | 15% | 7% | 12% |  |  |  |  |
| MDS9 | SMC6 | K456M | 2% | 18% | 16% | 16% |  |  |  |  |
| MDS9 | SP3 | D598N | 4% | 19% | 12% | 18% |  |  |  |  |
| MDS9 | TH | A159T | 15% | 5% | 4% | 5% |  |  |  |  |
| MDS17 | U2AF1 | Q84R | 52% | 29% |  |  |  |  |  |  |
| MDS17 | JAK2 | V617F | 42% | 39% |  |  |  |  |  |  |
| MDS17 | TET2 | R1366L | 5% | <1% |  |  |  |  |  |  |
| MDS17 | TET2 | E227fs | 51% | 40% |  |  |  |  |  |  |
| MDS17 | CRB2 | V359A | 33% | 0% |  |  |  |  |  |  |
| MDS17 | PTPRQ | M298V | 52% | NT |  |  |  |  |  |  |
| MDS17 | TP53 | P113fs | 5% | <1% |  |  |  |  |  |  |
| MDS20 | SF3B1 | K700E | 40% | 47% |  |  |  |  |  |  |
| MDS20 | MYH14 | P251A | 44% | 46% |  |  |  |  |  |  |
| MDS27 | SF3B1 | H662Q | 28% | 45% | 36% | 46% |  |  |  |  |
| MDS27 | ME1 | Y72H | 31% | 37% | 48% | 53% |  |  |  |  |
| MDS27 | CAMTA1 | C923F | 33.00% | 50% | 53% | 50% |  |  |  |  |
| MDS27 | PTPDC1 | A1640T | 21.00% | 39% | 35% | 44% |  |  |  |  |
| MDS37 | JAK2 | V617F | 4% | 3% | 1% |  |  |  |  |  |
| MDS37 | RUNX1 | Q268fs | 21% | 0% | 0% |  |  |  |  |  |
| MDS37 | SF3B1 | K700E | 39% | 49% | 50% |  |  |  |  |  |
| MDS37 | TET2 | F1041fs | 43% | 38% | 27% |  |  |  |  |  |
| MDS37 | TP53 | C199R | 2% | <1% | <1% |  |  |  |  |  |
| MDS38 | RBBP4 | K376N | 35% | 1% | 3% | 3% | 3% | 2% | <1% | 4% |
| MDS38 | RNF220 | R541Q | 25% | 4% | 2% | 3% | 5% | 1% | 0% | 7% |
| MDS38 | SEC31B | SS 1180-1G>A | 32% | 3% | 2% | 4% | 1% | 2% | 1% | 5% |
| MDS38 | TET2 | C1211Y | 26% | 1% | 3% | 1% | 3% | 2% | <1% | 6% |
| MDS38 | UBA1 | SS 2003+1->T | 70% | 1% | 6% | 2% | 3% | 6% | 5% | 15% |
| MDS38 | ZBTB33 | N134fs | 16% | 2% | 2% | 0% | 0% | 1% | 0% | 5% |
| MDS38 | ZNF318 | R1349X | 36% | 2% | 4% | 0% | 3% | 1% | 0% | 4% |
| MDS49 | DNMT3A | L347P | 35% | 2% | 30% | 29% |  |  |  |  |
| MDS49 | IRF2 | I60V | 45% | 24% | 37% | 32% |  |  |  |  |
| MDS49 | TET2 | R1452X | 6% | 0% | 1% | 6% |  |  |  |  |

**Supplementary Table 3:** Percentage of human cell engraftment in mice transplanted with CB CD34^+^ cells alone or with MSC^+^.

| Expt | Mouse Type | Cells Injected | CD34+ Injected | MSC Injected | hCD45 | CD19+ | CD33+ |
| --- | --- | --- | --- | --- | --- | --- | --- |
| 1 | NSG | CD34+MSC | 1x105 | 5x105 | 56 | 81.7 | 16.7 |
| 2 | NSG | CD34+MSC | 1x105 | 5x105 | 71.7 | 80.6 | 15.8 |
| 3 | NSG | CD34+MSC | 1x105 | 5x105 | 61.9 | 76.9 | 20.3 |
| 4 | NSG | CD34+MSC | 1x105 | 5x105 | 37.3 | 71.7 | 22.6 |
| 5 | NSG | CD34+MSC | 1x105 | 5x105 | 43.6 | 82.1 | 9.51 |
| 6 | NSG | CD34+MSC | 1x105 | 5x105 | 63.1 | 81.1 | 10.5 |
| 7 | NSG | CD34+MSC | 1x105 | 5x105 | 57.8 | 51.1 | 5.73 |
| 8 | NSG | CD34+MSC | 1x105 | 5x105 | 63.9 | 61.9 | 10.5 |
| 9 | NSG-S | CD34+MSC | 1x105 | 5x105 | 16.8 | 33.4 | 40.2 |
| 10 | NSG-S | CD34+MSC | 1x105 | 5x105 | 3.49 | 21.3 | 14.4 |
| 11 | NSG-S | CD34+MSC | 1x105 | 5x105 | 63 | 26.1 | 15.7 |
| 12 | NSG-S | CD34+MSC | 1x105 | 5x105 | 46.7 | 17.1 | 31.8 |
| 13 | NSG-S | CD34+MSC | 1x105 | 5x105 | 38.8 | 35.7 | 51.4 |
| 14 | NSG | CD34+ | 1x105 |  | 57.5 | 75.3 | 22 |
| 15 | NSG | CD34+ | 1x105 |  | 65.9 | 74.7 | 21.6 |
| 16 | NSG-S | CD34+ | 1x105 |  | 11.2 | 7.47 | 46.6 |
| 17 | NSG-S | CD34+ | 1x105 |  | 46.8 | 19.4 | 38.2 |

**Supplementary Table 4:** Somatic mutations detected at day0 and following LTC expansion on MSC or MS5 stroma.

| UPN | Gene Name | Amino Acid Change | Day0 MAB | LTC-IC MSC | LTC-IC MS5 |
| --- | --- | --- | --- | --- | --- |
| MDS47 | SF3B1 | K700E | 44% | 21% | 25% |
| MDS47 | ZFHX4 | G3230D | 46% | 43% | 40% |
| MDS47 | KRT8 | G440S | 38% | NT | NT |
| MDS47 | ACSM1 | R200X | 39% | 28% | 32% |
| MDS47 | TP53 | V83fs | 6% | 9% | BK |
| MDS48 | ATAD2B | N456D | 39.60% | NT | NT |
| MDS48 | ATG4B | SS 283+1G>A | 44.09% | NT | NT |
| MDS48 | CDK13 | SS 2898-2A>G | 9.78% | 21% | 12% |
| MDS48 | COG2 | Q694H | 9.42% | 2% | 9% |
| MDS48 | DNMT3A | Y735S | 48.60% | 35% | 46% |
| MDS48 | ENPP4 | N123S | 10.97% | NT | NT |
| MDS48 | PGBD1 | R48W | 32.87% | 29% | 37% |
| MDS48 | PXDN | G1255E | 41.42% | 50% | 37% |
| MDS48 | SF3B1 | K700E | 45.11% | 44% | 30% |
| MDS48 | TET2 | L1332fs | 7% | 3% | 1% |
| MDS48 | TET2 | C1221R | 9.67% | 9% | 2% |
| MDS48 | TET2 | Y1569X | 42.04% | 31% | 43% |
| MDS48 | ZZEF1 | F763fs | 43.10% | 26% | 34% |
| MDS44 | MPL | K553M | 44.44% | 48% | 48% |
| MDS44 | ACTL6A | P154S | 38.16% | 4% | 4% |
| MDS44 | IGF2BP2 | K446N | 48.25% | 55% | 52% |
| MDS44 | ADH1C | - | 98.25% | 94% | 99% |
| MDS44 | FTMT | R136Q | 45.26% | 58% | 56% |
| MDS44 | SLCO5A1 | I274M | 45.85% | 46% | 48% |
| MDS44 | MVK | V310M | 48.32% | 60% | 51% |
| MDS44 | RBM26 | T933fs | 50.00% | 50% | 42% |
| MDS44 | TRIM9 | V568A | 47.19% | 41% | 45% |
| MDS44 | SRSF2 | 95_103del | 38.30% |  | 18% |
| MDS44 | KRTAP6-2 | S58T | 41.67% | 51% | 49% |
| MDS44 | RUNX1 | A120fs | 29.51% | 25% | 26% |
| MDS44 | CHEK2 | R188W | 51.02% | 55% | 52% |
| MDS44 | FLNA | D1281E | 37.17% | 51% | 49% |
| MDS45 | CHPF | Q553X | 40.54% | 48% | 48% |
| MDS45 | TET2 | Q1191X | 38.73% | 33% | 43% |
| MDS45 | TET2 | I1873T | 31.73% | 42% | 43% |
| MDS45 | COL12A1 | G2987D | 42.86% | 42% | 47% |
| MDS45 | ANK1 | K6X | 27.36% | 36% | 48% |
| MDS45 | LRIT2 | Q254H | 31.15% | 21% | 21% |
| MDS45 | CYP2C18 | D143G | 37.84% | 41% | 46% |
| MDS45 | POLE | R1909Q | 30.30% | 53% | 59% |
| MDS45 | ZRSR2 | T232fs | 47.69% | 42% | 46% |
| MDS43 | ASXL1 | L823X | 40.48% | 41% | 51% |
| MDS43 | RUNX1 | S341PfsX253 | 39.79% | 34% | 46% |
| MDS49 | DNMT3A | L347P | 35.22% | 30% |  |
| MDS49 | DNMT3A | R899C | 6.00% |  |  |
| MDS49 | IRF2 | I60V | 45.45% | 37% |  |
| MDS49 | SF3B1 | E622D | 30.33% | 32% |  |
| MDS49 | TET2 | R1452X | 5.93% | 4% |  |

**Supplementary Table 5:** Number of hCD45+ cells recovered following LTC.

| UPN | WHO Diagnosis | Stroma Type | hMSC/MS5 | | CD34+ Plated | hCD45 Recovered total |
| --- | --- | --- | --- | --- | --- | --- |
| MDS47 | RARS | Auto | 5x104 | | 2x103 | 1.4x105 |
| MDS47 | RARS | MS5 | | 5x104 | 2x103 | 2.8x105 |
| MDS48 | RARS | Auto | | 5x104 | 2x103 | 3.3x105 |
| MDS48 | RARS | MS5 | | 5x104 | 2x103 | 3.34x105 |
| MDS49 | RARS | Auto | | 5x104 | 2x103 | 7.4x105 |
| MDS45 | RCMD | Auto | | 5x104 | 2x103 | 1.5x105 |
| MDS45 | RCMD | MS5 | | 5x104 | 2x103 | 2.5x105 |
| MDS46 | RCMD | Auto | | 5x104 | 2x103 | 2.3x105 |
| MDS46 | RCMD | MS5 | | 5x104 | 2x103 | 3x105 |
| MDS44 | RAEB | Auto | | 5x104 | 2x103 | 1.7x104 |
| MDS44 | RAEB | MS5 | | 5x104 | 2x103 | 2.1x105 |
| MDS43 | RAEB | Auto | | 5x104 | 2x103 | 1.6x104 |
| MDS43 | RAEB | MS5 | | 5x104 | 2x103 | 1x105 |
| MDS22 | RCMD | Allo RARS | | 5x104 | 2x103 | 3.4x105 |
| MDS42 | RCMD | Allo RARS | | 5x104 | 2x103 | 2.6x106 |

**References**

1. Mian SA, Rouault-Pierre K, Smith AE, Seidl T, Pizzitola I, Kizilors A*, et al.* SF3B1 mutant MDS-initiating cells may arise from the haematopoietic stem cell compartment. *Nat Commun* 2015 Dec 08; **6:** 10004
2. Mohamedali AM, Gaken J, Ahmed M, Malik F, Smith AE, Best S, et al. High concordance of genomic and cytogenetic aberrations between peripheral blood and bone marrow in myelodysplastic syndrome (MDS). Leukemia. 2015; 29(9):1928-38.
